# Helical Phase Structure of Radiation from an Electron in Circular Motion

**DOI:** 10.1038/s41598-017-06442-2

**Published:** 2017-07-21

**Authors:** M. Katoh, M. Fujimoto, N. S. Mirian, T. Konomi, Y. Taira, T. Kaneyasu, M. Hosaka, N. Yamamoto, A. Mochihashi, Y. Takashima, K. Kuroda, A. Miyamoto, K. Miyamoto, S. Sasaki

**Affiliations:** 1 Institute for Molecular Science, National Institutes of Natural Sciences, Okazaki, 444-8585 Japan; 2 Sokendai (The Graduate University for Advanced Studies), Okazaki, 444-8585 Japan; 3 National Institute of Advanced Industrial Science and Technology (AIST), Tsukuba, 305-8565 Japan; 4 Saga Light Source, Tosu, 841-0005 Japan; 5 Nagoya University, Nagoya, 464-0814 Japan; 6 University of Tokyo, Kashiwa, 277-0882 Japan; 7 Hiroshima University, Higashi-Hiroshima, 739-0046 Japan

## Abstract

We theoretically show that a single free electron in circular motion radiates an electromagnetic wave possessing helical phase structure, which is closely related to orbital angular momentum carried by it. We experimentally demonstrate it by interference and double-slit diffraction experiments on radiation from relativistic electrons in spiral motion. Our results indicate that photons carrying orbital angular momentum should be created naturally by cyclotron/synchrotron radiations or Compton scatterings in various situations in cosmic space. We propose promising laboratory vortex photon sources in various wavelengths ranging from radio wave to gamma-rays.

## Introduction

Twisted photons possessing helical wave front carry orbital angular momentum other than well-known spin angular momentum^1^. Such photons have been investigated theoretically and experimentally, in particular, towards applications in the information technologies, the nanotechnologies and the imaging technologies^2, 3^. Their interactions with nuclei, atoms, molecules, materials or plasma have been also being explored theoretically rather than experimentally^2–4^, that might be due to the lack of the laboratory photon sources other than in the laser wave length range where twisted photon beams can be readily obtained by using conventional optical devices. Twisted photons in nature, particularly in astrophysics, have been reviewed^5, 6^. However, the authors did not discuss the sources of twisted photons explicitly because they could not find promising radiation processes. Instead, they discussed detection schemes of twisted photons in astronomical observations. Other authors proposed twisted radiation from rotating black holes or inhomogeneous interstellar media^7, 8^, however, they discussed modification of radiation rather than radiation processes itself.

In this paper, we show that a single free electron in circular motion emits twisted photons. This is one of the most fundamental radiation processes by a free electron and is the basis of synchrotron/cyclotron radiations or Compton scattering, which play important roles in various situations in nature or in laboratories, such as around magnetized neutron stars, in supernova explosions with magnetic fields, in nuclear fusion plasma, electron accelerators and so on. The radiations from such electrons cover the entire range of wave lengths, from radio wave to gamma-rays, depending on the physical parameters such as the electron energy or radius of motion. They possibly play important roles with their angular momentum in nature and in laboratories, which have not been recognized by researchers. Moreover, this radiation process can be the basis of laboratory vortex photon sources in the entire wavelength range, which will open new research opportunities.

Radiation generated by electrons in circular motion was first studied by O. Heaviside in 1904^9^ and has since been addressed in many scientific reports^10, 11^ and textbooks^12, 13^. In the following, we derive an analytic expression that contains a term representing the spatial phase structure explicitly. We show that the radiation field possesses a spiral phase structure. We explain intuitively the mechanism which produces the structure.

**Figure 1:**
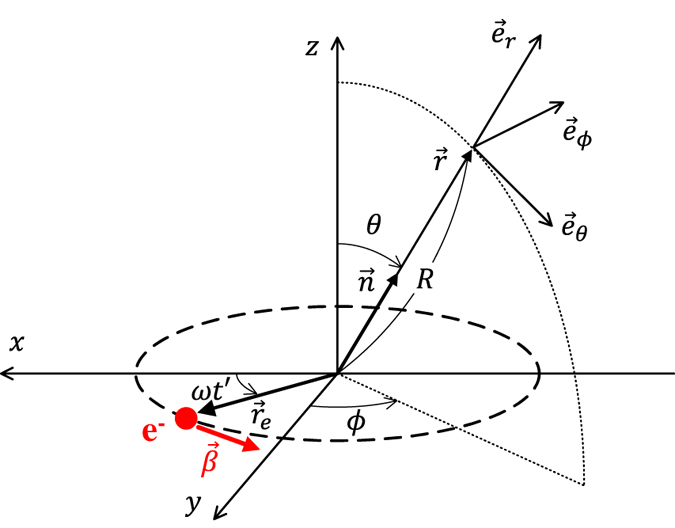
Coordinate system. The electron is rotating in the x-y plane around the origin with an initial position on the x-axis. The azimuthal angle of the position of the observer is measured from the y-axis. The observer frame is defined as a spherical coordinate.

We assume that the electron trajectory draws a circle in a plane perpendicular to the z-axis as shown in Fig. 1. The radiation field is given by the following formulae, which are directly derived from the Liénard–Wiechert potentials^12^:

1 $$\overrightarrow{E}(t)={\frac{e}{cR}\frac{\overrightarrow{n}\times \{(\overrightarrow{n}-\overrightarrow{\beta })\times \dot{\overrightarrow{\beta }}\}}{{(1-\overrightarrow{n}\cdot \overrightarrow{\beta })}^{3}}|}_{t\text{'}}$$

2 $$\overrightarrow{H}(t)={\overrightarrow{n}\times \overrightarrow{E}|}_{t\text{'}}$$

3 $$t\text{'}=t-\frac{R}{c}$$

Here, $\overrightarrow{E}(t)$ and $\vec{\mathop{H}\limits^{\rightharpoonup }}(t)$ are the electric and magnetic fields, respectively, *c* is the speed of light, *e* is the elemental charge, $\overrightarrow{n}$ is the unit vector, *R* is the distance from the origin to the observer (see Fig. 1), $\overrightarrow{\beta }$ is the electron velocity normalised by the velocity of light, and *ω* is the electron angular velocity. The right sides of Eqs (1) and (2) represent the emitter time $t\text{'}$, which is related to the observer time *t* by Eq. (3). An expression for the electric field in the observer frame is obtained by inserting adequate expressions for the vectors in Eq. (1):

4 $$\overrightarrow{E}(R,\theta ,\phi ,t)=\frac{e}{cR}\frac{\beta \omega }{{\{1-\beta \sin \theta \cos (\omega t\text{'}-\phi )\}}^{3}}[\cos \,\theta \,\sin \,(\omega t\text{'}-\phi ){\overrightarrow{e}}_{\theta }-\{\cos (\omega t\text{'}-\phi )-\beta \,\,\sin \,\theta \}{\overrightarrow{e}}_{\phi }]$$

Because the magnetic field can be obtained using Eq. (2), we do not show its explicit form.

**Figure 2:**
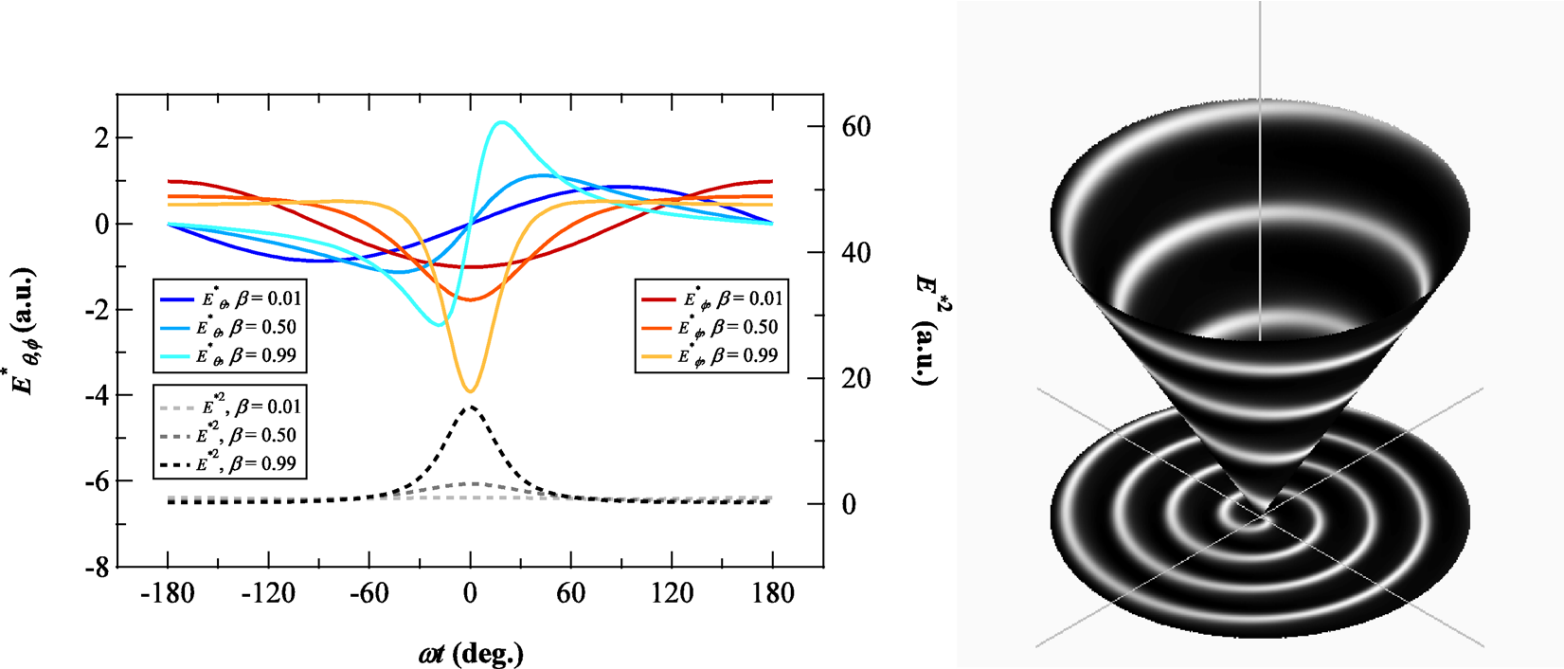
Left: Waveform of the electric field propagating towards the polar angle (*θ*) of 30° and the azimuthal angle (*ϕ*) of 0° for a range of electron velocities, *β*. Blue lines represent the *θ* components, and red lines represent the *ϕ* components (see Fig. 1). Black dotted lines show the field intensities, given as the square summation of the electric field components. To emphasise the change in the waveform, the electric fields are divided by *β* in the calculation (see Eq. (4)). Right: Radiation field intensity propagating towards 30° from the z-axis and its projection on the x-y plane. The electron velocity *β* is 0.5. The brightness is the magnitude.

The waveforms of the radiation fields calculated using Eq. (4) are shown in Fig. 2. As the electron velocity increases, the sinusoidal waveforms become distorted and develop kinks around the phase, corresponding to the time when the instantaneous electron motion is directed to the observer (Fig. 1). The deformed wave can be decomposed into harmonic components with frequencies that are integer multiples of ω^9^.

**Figure 3:**
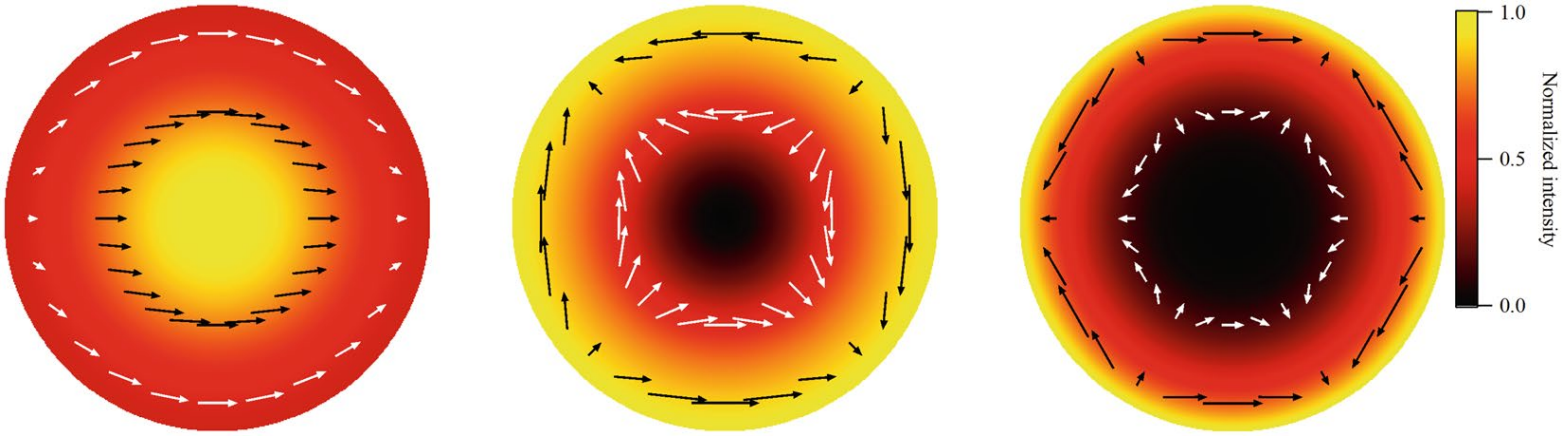
Electric field distribution in the upper hemisphere viewed from the z-direction (see Fig. 1), from left to right, for the fundamental (*l* = 1), second (*l* = 2) and third (*l* = 3) harmonics calculated from Eq. (5). The colour represents the field intensity. The fundamental frequency has an intensity maximum in the centre, whereas the harmonics show zero intensity at the centre. Arrows represent the direction of the electric field at a specific time.

The spatial distribution of the propagating electric field is also shown in Fig. 2. The relativistic kink, where the field is strengthened, is distributed as a spiral. This suggests that the phase of the higher harmonic components also depends on the azimuthal angle. Such a behavior of the radiation field can be seen in the textbooks describing synchrotron radiation^13, 14^. However, the authors did not discuss it in the context with the phase structure or the angular momentum. We decompose Eq. (4) into a Fourier series to demonstrate the spiral phase structure analytically, following the previous literature^12^, and we express the result by unit vectors in Cartesian coordinates to make the phase structure explicit:

5 $$\begin{array}{rcl}\overrightarrow{E}({R}_{0},\theta ,\phi ,\omega t) & = & {\rm{Re}}\sum _{l=1}^{\infty }\frac{e}{cR}l\omega \{{\varepsilon }_{+}^{l}(\theta ){e}^{i(l-1)\phi }{\overrightarrow{e}}_{+}+{\varepsilon }_{-}^{l}(\theta ){e}^{i(l+1)\phi }{\overrightarrow{e}}_{-}+i{\varepsilon }_{z}^{l}(\theta ){\overrightarrow{e}}_{z}{e}^{il\phi }\}{e}^{-il(\omega t-\frac{{R}_{0}}{c})}\\ \quad \quad \,\,\,{\varepsilon }_{\pm }^{l}(\theta ) & \equiv  & \frac{{\varepsilon }_{x}^{l}(\theta )\pm {\varepsilon }_{y}^{l}(\theta )}{\sqrt{2}}\\  & = & \beta {J}_{l}^{^{\prime} }(l\beta \,\sin \,\theta )\pm \frac{{\cos }^{2}\,\theta }{\sin \,\theta }{J}_{l}(l\beta \,\sin \,\theta )\\ \quad \quad \quad \,\,\,{\varepsilon }_{z}^{l} & \equiv  & \cos \,\,\theta {J}_{l}(l\beta \,\sin \,\theta )\end{array}$$

where *J*_*l*_ and ${J}_{l}\text{'}$ are a Bessel function of the first kind and its derivative, respectively. Here, we have introduced rotation vectors related to the unit vectors in the x and y directions as ${\overrightarrow{e}}_{\pm }=({\overrightarrow{e}}_{x}\pm i{\overrightarrow{e}}_{y})/\sqrt{2}$. The first term in the parentheses represents circular polarised components with the same helicity as that of the electron motion, and the second term represents components with the reverse helicity. The third term arises from the spherical nature of the field. The summation of these three terms represents the elliptical polarisation. The electric fields of the fundamental, second and third harmonics calculated from Eq. (5) are shown in Fig. 3. The vortex nature is clearly observed in the electric field distribution of the second and third harmonics but not in the electric field distribution of the fundamental component.

In Eq. (5), when *θ* is small, the first term becomes dominant and the field is accurately represented by paraxial approximation. Its polarisation is circular and has a phase term $\exp \,\{i(l-1)\varphi \}$ that is a common feature of a vortex beam. According to Allen *et al*.^1^, such a field possesses an orbital angular momentum of *l*−1. In the general case of *θ*, the field represented by Eq. (5) is non-paraxial. It has been argued that the radiation field of such a case possesses angular momentum, even though the spin and orbital angular momentum are difficult to separate^15^. Recently we have successfully derived an expression for the ratio of the angular momentum density to the energy density for the radiation emitted by an electron in circular motion in a separate paper, taking a different mathematical approach^16^. The expression shows that a photon of the *l*-th harmonic carries the total angular momentum whose z-component is equal to *l*. It may be interpreted as that the first component in Eq. (5) has spin angular momentum of +1 and orbital angular momentum of *l*−1, whereas the spin and orbital angular momenta of the second component are −1 and *l* + 1, respectively. The total angular momentum in the z direction is always *l*.

When an electron in circular motion drifts along the *z*-axis with high relativistic velocity, the radiation field perpendicular to the *z*-axis is strengthened by the Lorentz factor *γ*, which is given by the electron energy divided by the electron rest mass energy, and is collimated into a narrow cone around the *z*-axis^12^. Consequently, the field is well represented by the paraxial approximation. Such radiation fields can be produced in the laboratory using a helical undulator. These devices are widely used as synchrotron light sources^17^, wherein a high-energy electron beam executes spiral motion in a specially designed magnetic field, radiating circularly polarised light. This has been identified as a vortex photon source both mathematically^18^ and experimentally^19^, although the origin of the twisting has not been addressed. We acknowledge that these pioneering works inspired us to carry out this work. The helical undulator radiation corresponds to the case where an electron in circular motion travels towards the *z*-axis at relativistic velocity. Because the phase is Lorentz invariant, the harmonic components of the helical undulator radiation should preserve the helical phase structure, which is consistent with the conservation of angular momentum along the direction of motion of the frame in the Lorentz transformation^12^.

Here, we present experimental results that provide clear evidence of the twisted nature of the harmonics and the non-vortex nature of the fundamental as expressed by Eq. (5). To observe the spatial phase structure, a significant fraction of the radiation field should be observed simultaneously. The collimated nature of the undulator radiation provides an ideal experimental condition. We have carried out a series of experiments on the optical vortex beam from an undulator at the UVSOR-III electron storage ring^20, 21^. Some relevant results are shown below.

**Figure 4:**
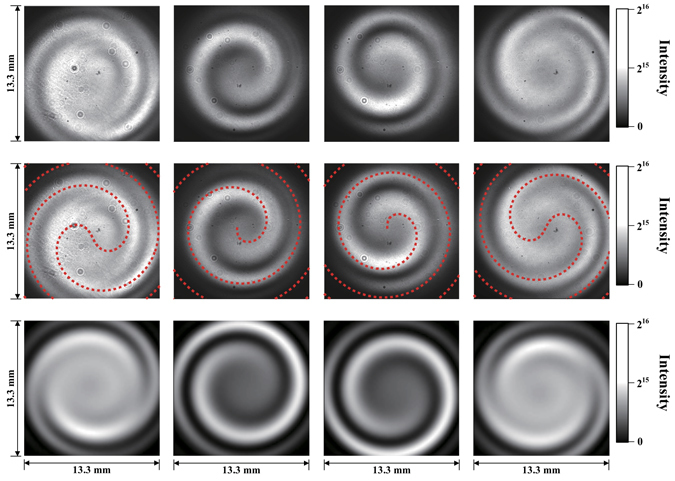
Interference between two undulator radiations. From the left column to the right: interference patterns between the fundamental and third harmonics and between the fundamental and second harmonics for left-handed polarisation, and between the fundamental and second harmonics and between the fundamental and third harmonics for right-handed polarisation. From the top row to the bottom, the raw CCD images, those with the analytic calculation results (red dotted lines) following Bahrdt *et al*.^19^ and the numerical simulation results by SRW^25^. The handedness is defined along the electron beam direction. The centres of the analytic results are fitted to the measurements.

First, we discuss the interference between the fundamental radiation and harmonics. The interference measurement between a reference beam and a vortex beam is an established method to show its phase structure^22^. Bahrdt *et al*. proposed a novel technique to apply this method for undulator radiation and successfully demonstrated it for the fundamental and second harmonics^19^. Following their approach, we carried out interference experiment including the higher harmonics. Figure 4 shows the interference patterns between the fundamental radiation from one undulator and the second or third harmonics from another undulator. They are compared with analytic calculations based on the formulae in the references^19, 23^ and a simulation code SRW^24^. Single- and double-spiral structures are clearly observed and they are well reproduced by the analytic calculations and the numerical simulations. This result clearly shows that the orbital angular momentum increases as the harmonic number increases, which is one of the important prediction of Eq. (5). Moreover, the directions of the spiral structures are reversed with the reversal of the electron circulation direction. These results indicate that the relative phase difference between the light beams is in agreement with the theoretical prediction of Eq. (5). However, at this stage, the absolute phase structure of radiation generated by a single undulator is unclear.

**Figure 5:**
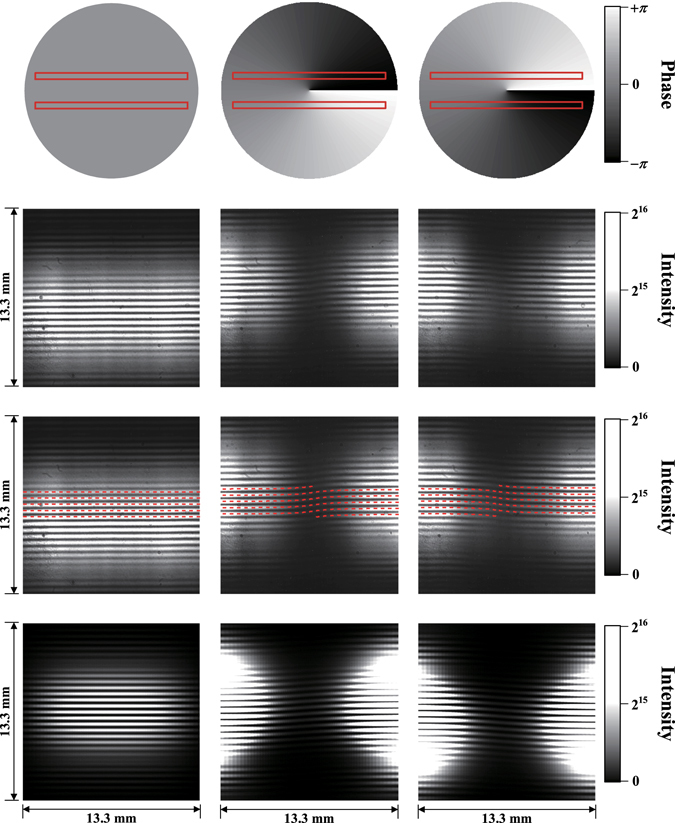
Double-slit diffraction patterns of undulator radiation. From the left column to the right; the fundamental, the second harmonic (left-handed) and the second harmonic (right-handed), from the top row to the bottom; schematic drawings of the double-slits (red rectangles) and the phase distributions, the raw CCD camera images, those with the analytic calculation following Sztul and Alfano^24^ (red dotted lines) and the SRW simulation results^25^. The handedness is defined as same as in Fig. 4. The centres of the analytic calculation results are fitted to the measurements.

Next, we present the results from a double-slit diffraction experiment. This is the first experimental result to demonstrate the vortex nature of radiation from a single undulator using longitudinally incoherent but spatially coherent radiation. The fundamental and second harmonic components in the ultraviolet range were extracted and irradiated onto a double-slit apparatus. The diffraction patterns were observed using a charge-coupled device (CCD) camera, as shown in Fig. 5. An ordinary stripe pattern was observed for the fundamental radiation, as expected for plane waves. However, for the second harmonic, we observed a singularity in the middle of the pattern, as has been demonstrated for a Laguerre–Gaussian beam created by a laser^25^. This singularity arises from the change in the phase difference between the two slits, as illustrated in Fig. 5. The singularity appeared only when the centre of the beam was located between the slits. The observed patterns were accurately reproduced by the analytic expression^25^ and SRW code^24^. We noted that diffraction experiments were carried out for coherent vortex radiation from a single undulator^26, 27^. However, these experiments are inadequate to show the property of radiation from a single electron, because, in case of coherent radiation, the radiation field is strongly affected by the distribution of electrons^26, 28^. We also noted that a vortex X-ray beam was successfully produced by using spiral phase plate and undulator beam, which may be an alternative approach for practical applications^29^.

We have shown that the harmonic components of an electromagnetic field radiated by electrons in circular motion naturally have a helical phase structure, which suggests the presence of orbital angular momentum. We demonstrated this experimentally by observing helical undulator radiation. The generation of a twisted photon beam from a helically micro-bunched electron beam was reported^27^. However, a vortex radiation field was formed by the constructive interference of the non-twisted radiation from the electrons that were helically aligned in space. This is analogous to the generation of monochromatic radiation from a micro-bunched beam travelling in a uniform magnetic field, even though each electron produces broadband synchrotron radiation^28^.

This work allows us to predict the conditions under which twisted radiation will be produced. We propose that cyclotron/synchrotron radiation, particularly from an electron cyclotron maser^30^, should be re-examined as twisted radiation. Another candidate is the non-linear inverse Compton scattering of circular-polarised light. In this case, the intense incoming light field causes relativistic circular motion of electrons, thereby producing twisted harmonic radiation, as described in a separated paper^31^. These radiation processes may play important, unexplored roles in the solar magnetosphere^10^ or planetary magnetosphere^32^, around magnetised neutron stars^33^, around active galactic nuclei^34^, in tokamaks used for nuclear fusion^35^, or in particle accelerators^17^. Observations using phase information may provide new approaches to the analysis of such systems. Several methods have been proposed for detecting vortex photons in nature, but there has been no explicit discussion of the sources of such radiation^5, 6, 36^. Our work suggest some possible vortex photon sources in nature.

This work also suggests possible technologies for producing twisted photon beams in laboratories at wavelengths ranging from radio waves to gamma rays. In the ultraviolet and X-ray ranges, helical undulators can provide high-brightness twisted radiation. In the microwave and terahertz ranges, gyrotrons, where high-energy electrons execute circular motion and produce cyclotron radiation with harmonics, are potentially powerful sources of twisted radiation^37^. Another candidate is the non-linear inverse Compton scattering of intense circular-polarised laser radiation by a relativistic electron beam provided by an accelerator, which could potentially provide a twisted X-ray or gamma-ray source^31^. The development of these light sources will expand the application of twisted radiation to the entire electromagnetic wavelength range where alternative methods^38–40^ are not applicable.

## Methods

This experiment was conducted at the BL1U beamline of the UVSOR-III electron storage ring, which is equipped with two polarisation-variable undulators in tandem. The undulators had 10 magnetic periods with an 88-mm period length. Two undulators are separated by a space of approximately 0.5 m. The undulators were operated in circular-polarised mode during the experiments. In the undulators, the electron beam executes spiral motion. We define the handedness of electron circulation and light polarisation along the beam direction, such that clockwise circulation around the beam axis is right-handed. The electrons produce quasi-monochromatic synchrotron radiation and harmonics in the ultraviolet wavelength range. This major advantage of our experiment allowed us to perform all of the experiments in the air using ordinary optical components and devices. The UVSOR-III electron storage ring was operated at 500 MeV for the experiment including up to 2^nd^ harmonic and 400 MeV for those including up to 3^rd^ harmonic, which are lower than the nominal electron energy of 750 MeV. The electron beam emittance at this energy was estimated to be 8 nm-rad and 5nm-rad, respectively. The electron beam was diffraction-limited in the UV wavelength range; therefore, the undulator radiation was spatially coherent, which was essential for the experiments described below. The typical electron beam current used in the experiment was 1 mA, much smaller than the normal operating current but sufficient for these measurements. The undulator radiation was extracted from the accelerator through the SiO_2_ window, without the use of mirrors or monochromators.

**Figure 6:**
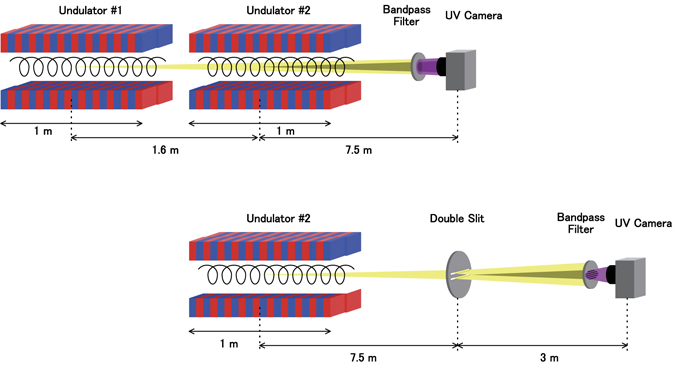
Experimental setup of the interference experiment (upper) and the double-slit diffraction experiment (lower). Electrons travel from left to right while executing spiral motion.

In the interference experiment, two undulators in tandem were tuned to produce either the fundamental radiation or harmonics at the same wavelength, i.e., 355 nm. The two interfered light beams were observed via a CCD camera (BITRAN BK50-UV) with a bandpass filter at 355 nm and a width of 1.3 nm, which was positioned approximately 7.5 m downstream of the centre of the second undulator and was directly viewing the undulators. The CCD is 13.3 mm × 13.3 mm in size with 1024 × 1024 pixels. In the diffraction experiments, a double slit with a width of 200 microns and separation of 2 mm was positioned approximately 7.5 m from the centre of the helical undulator and was aligned so that the optical beam centre was in the middle of the slits. The diffraction image was observed via the same CCD camera as in the interference experiment with a 355-nm bandpass filter placed 3 m downstream from the slit. A diffraction pattern with a singularity, as shown in Fig. 6, appeared when the beam centre was located between the slits but not when the slit position was shifted vertically by 3 mm in either direction. To obtain a clear diffraction pattern for the images presented in this paper, a linear polarised filter was placed in the vertical direction to eliminate contamination from the horizontally polarised radiation caused by the bending magnets. Neutral density (ND) filters were added as necessary to prevent saturation of the CCD camera.

## References

[1] Allen L, Beijersbergen MW, Spreeuw RJC, Woerdman JP (1992). Orbital angular momentum of light and the transformation of Laguerre-Gaussian laser modes. Phys. Rev. A..

[2] Torres, J. P. & Torner L. (eds.) *Twisted Photons*; *Application of Light with Orbital Angular Momentum*, WILEY-VCH Verlag GmbH & Co. KGaA (2011).

[3] Molina-Terriza G, Torres JP, Torner L (2007). Twisted photons. Nat. Phys..

[4] van Veenendaal M, McNulty I (2007). Prediction of strong dichroism induced by x rays carrying orbital momentum. Phys. Rev. Lett..

[5] Harwit M (2003). Photon orbital angular momentum in astrophysics. Astrophys. J..

[6] Elias NM (2008). Photon orbital angular momentum in astronomy. Astron. & Astrophys..

[7] Gray MD, Pisano G, Maccalli S, Schemmel P (2014). Amplification of OAM radiation by astrophysical masers. Mon. Not. R. Astron. Soc..

[8] Tamburini F, Thidé B, Molina-Terriza G, Anzolin G (2011). Twisting of light around rotating black holes. Nat. Phys..

[9] Heaviside O (1904). The Radiation from an Electron describing a Circular Orbit. Nature..

[10] Takakura T (1967). Theory of solar bursts. Solar Phys..

[11] Schwinger J (1949). On the Classical Radiation of Accelerated Electrons. Phys. Rev..

[12] Landau, L. D. & Lifshitz, E. M. *The classical theory of fields*. (4th Rev. English Ed.), Elsevier Ltd (1975).

[13] Jackson, J. D. *Classical Electrodynamics*. (3rd ed.) John Wiley & Sons, Inc (1999).

[14] Hofmann, A. *The Physics of Synchrotron Radiation*, Cambridge Univ. Press (2004).

[15] Barnett SM, Allen L (1994). Orbital angular momentum and nonparaxial light beams. Opt. Comm..

[16] Katoh M (2017). Angular Momentum of Twisted Radiation from an Electron in Spiral Motion. Phys. Rev. Lett..

[17] Winick, H. (sd.) *Synchrotron Radiation Sources; A Primer*. World Scientific Publishing Co. Pte Ltd (2014).

[18] Sasaki S, McNulty I (2008). Proposal for generating brilliant x-ray beams carrying orbital angular momentum. Phys. Rev. Lett..

[19] Bahrdt J (2013). First observation of photons carrying orbital angular momentum in undulator radiation. Phys. Rev. Lett..

[20] Sasaki, S. *et al*. Analyses of Light’s Orbital Angular Momentum from Helical Undulator Harmonics. *Proc. IPAC2015*, 1563–1566, *The Joint Accelerator Conferences Website (JACoW)* http://accelconf.web.cern.ch/AccelConf/IPAC2015/papers/tupwa061.pdf, (Date of access:28/03/2017) (2015).

[21] Hosaka, M. *et al*. Experimental Study on Optical Vortex from a Helical Undulator at UVSOR-III, *Proc. IPAC2016*, 2036–2038, *The Joint Accelerator Conferences Website (JACoW)* http://accelconf.web.cern.ch/AccelConf/ipac2016/papers/weoaa03.pdf (Date of access:28/03/2017) (2016).

[22] Vickers, J. *et al*. Phase and interference properties of optical vortex beams. *J. Opt. Soc. Am*. A **823**, 25, 3, 823–827 (2008). 10.1364/josaa.25.000823 18311255

[23] Bahrdt, J. *et al*. Undulator Photon Beams with Orbital Angular Momentum. *Proc. IPAC2014*, 213–215, *The Joint Accelerator Conferences Website* (*JACoW*) http://accelconf.web.cern.ch/AccelConf/IPAC2014/papers/mopro057.pdf, (Date of access:28/03/2017) (2014).

[24] Chubar O (1999). Phase analysis and focusing of synchrotron radiation. Nucl. Instr. Meth. Phys. Res. A.

[25] Sztul HI, Alfano RR (2006). Double-slit interference with Laguerre-Gaussian beams. Opt. Lett..

[26] Hemsing E (2014). Phys. Rev. Lett..

[27] Hemsing E (2013). Coherent optical vortices from relativistic electron beams. Nat. Phys..

[28] Bielawski S (2008). Tunable narrowband terahertz emission from mastered laser–electron beam interaction. Nat. Phys..

[29] Peele, A. G. *et al*. X-ray phase vortices: theory and experiment. *J. Opt. Soc. Am*. A 21, **8**, 1575–1584 (2004). 10.1364/josaa.21.001575 15330486

[30] Treumann RA (2006). The electron–cyclotron maser for astrophysical application. Astron. Astrophys. Rev..

[31] Taira, Y., Hayakawa, T., Katoh, M. Gamma ray vortices from nonlinear inverse Compton scattering of circularly polarized light. *arXiv:1608.04894v1* [*physics.acc-ph*] https://arxiv.org/abs/1608.04894, (Date of Access:01/06/2017) (2016). 10.1038/s41598-017-05187-2 PMC5504041 28694458

[32] de Pater I (1983). Observations and models of the decimetric radio emission from Jupiter. Adv. Space Res..

[33] Ginzburg VL, Syrovatskii SI (1965). Cosmic magnetobremsstrahlung (synchrotron radiation). Annu. Rev. Astron. Astrophys..

[34] Krawczynski H, Treister E (2013). Active galactic nuclei–the physics of individual sources and the cosmic history of formation and evolution. Front. Phys..

[35] Bornatici M (1983). Electron cyclotron emission and absorption in fusion plasmas. Nucl. Fusion.

[36] Berkhout GCG, Beijersbergen MW (2008). Method for probing the orbital angular momentum of optical vortices in electromagnetic waves from astronomical objects. Phys. Rev. Lett..

[37] Kartikeyan, M. V. *et al*. *Gyrotrons: High-Power Microwave and Millimeter Wave Technology*. (Advanced Texts in Physics) Springer (2004).

[38] Naidoo D (2016). Controlled generation of higher-order Poincaré sphere beams from a laser. Nat. Photonics.

[39] Rego L (2016). Nonperturbative Twist in the Generation of Extreme-Ultraviolet Vortex Beams. Phys. Rev. Lett..

[40] Hernández-García C (2013). Attosecond Extreme Ultraviolet Vortices from High-Order Harmonic Generation. Phys. Rev. Lett..

